# Supplementary figures and images for: Bounded rational decision-making models suggest capacity-limited concurrent motor planning in human posterior parietal and frontal cortex
Source: PLoS Comput Biol. 2022 Oct 13;18(10):e1010585. doi: 10.1371/journal.pcbi.1010585 (PMC9560147; doi:10.1371/journal.pcbi.1010585)

PMdl [-21.8,-6,57.5]

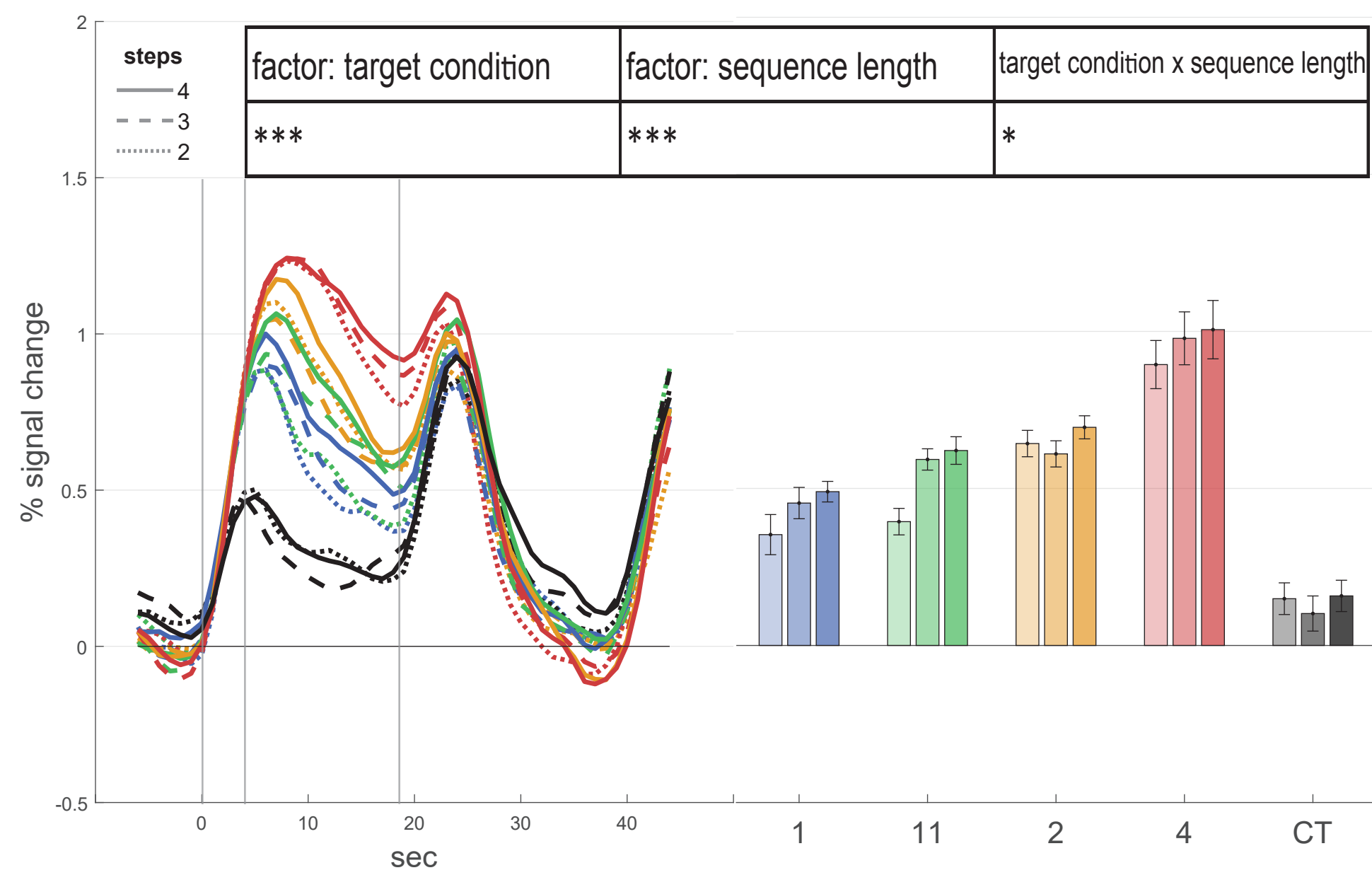

antlPSI [-36.3,-43.6,44.7]

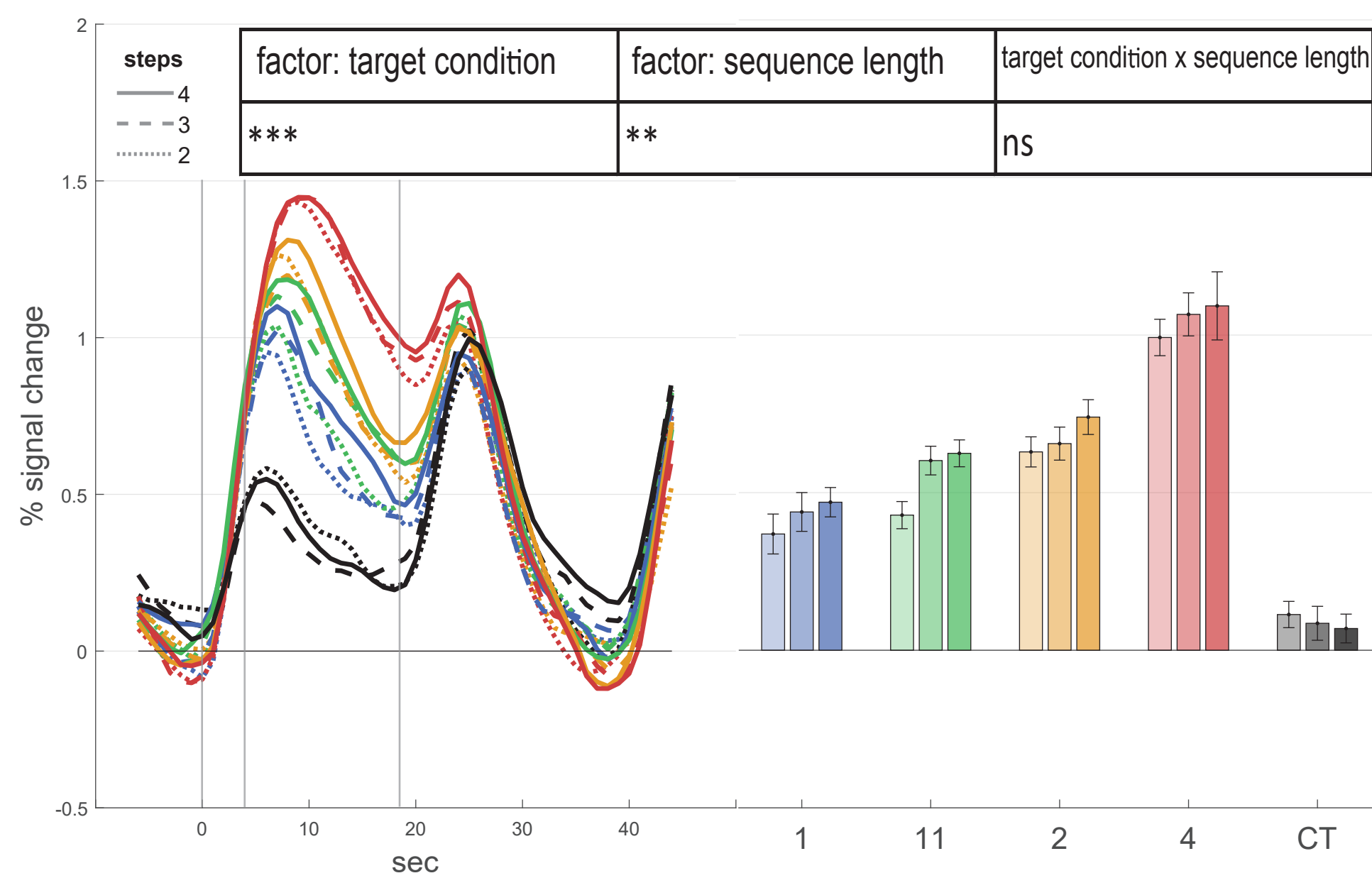

DLPFCI [-38.4,30.2,27.8]

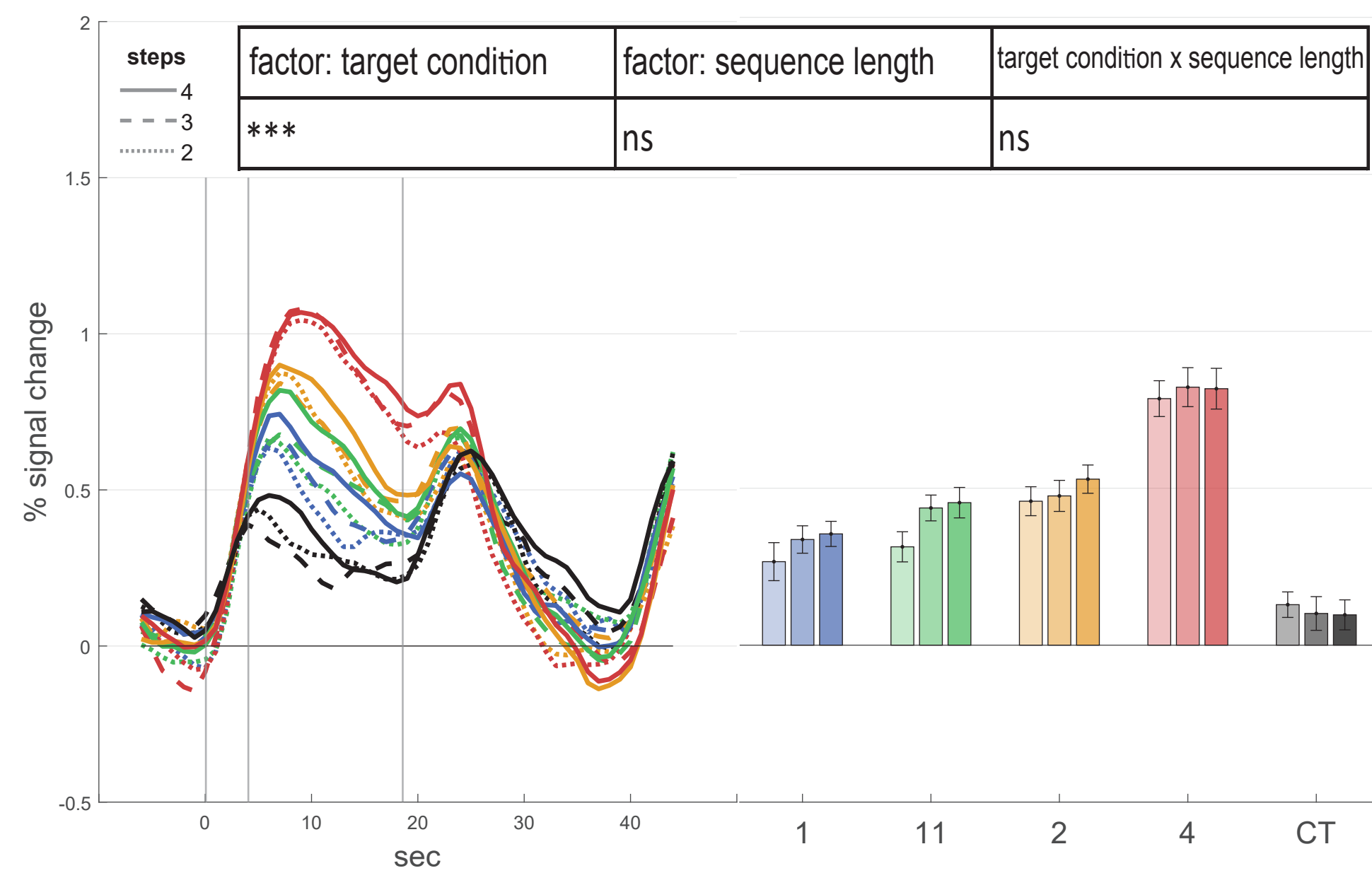

AICI [-32.7,19.6,4.6]

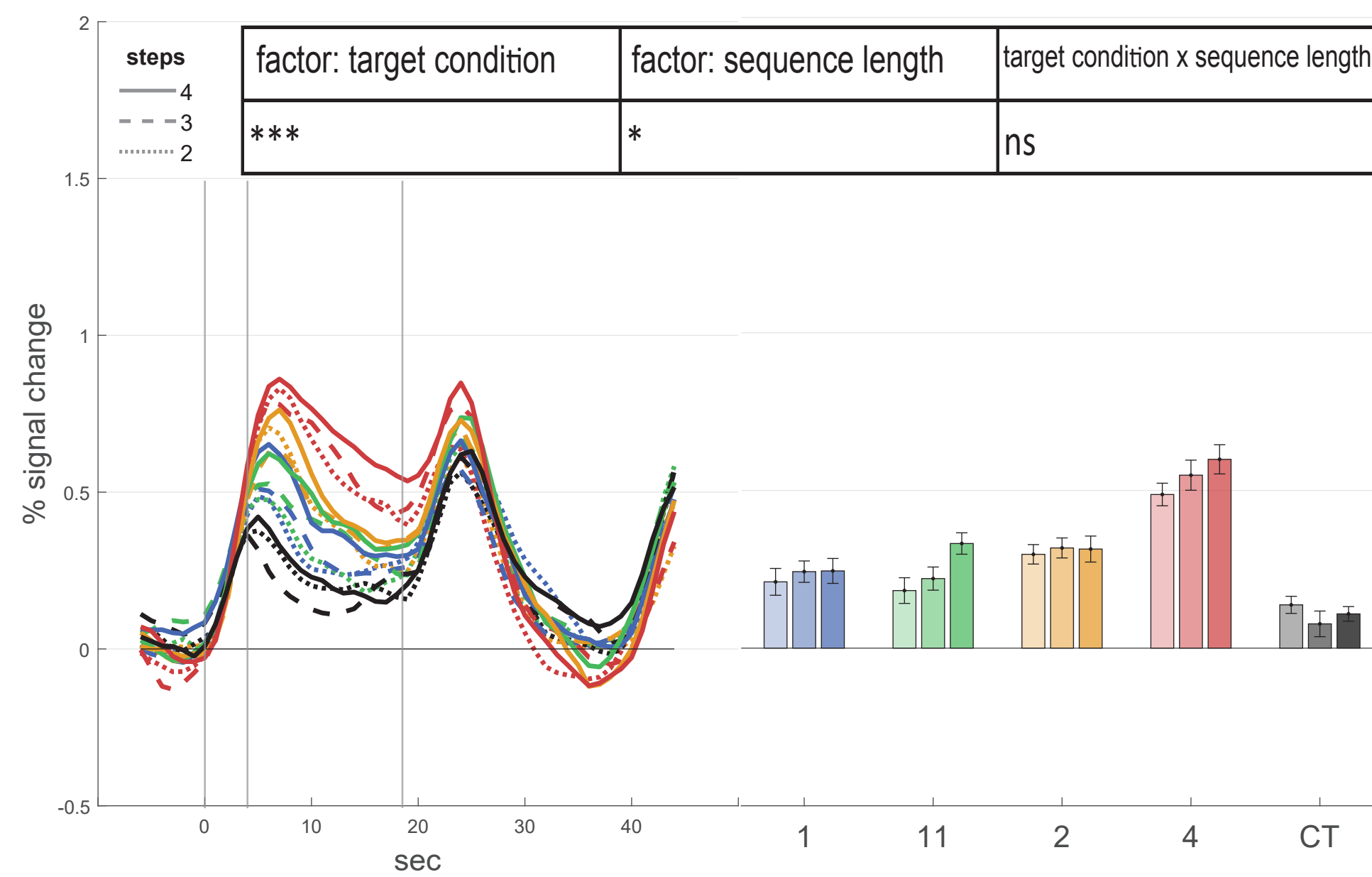

cer6r [31.9,-57.8,-29.7]

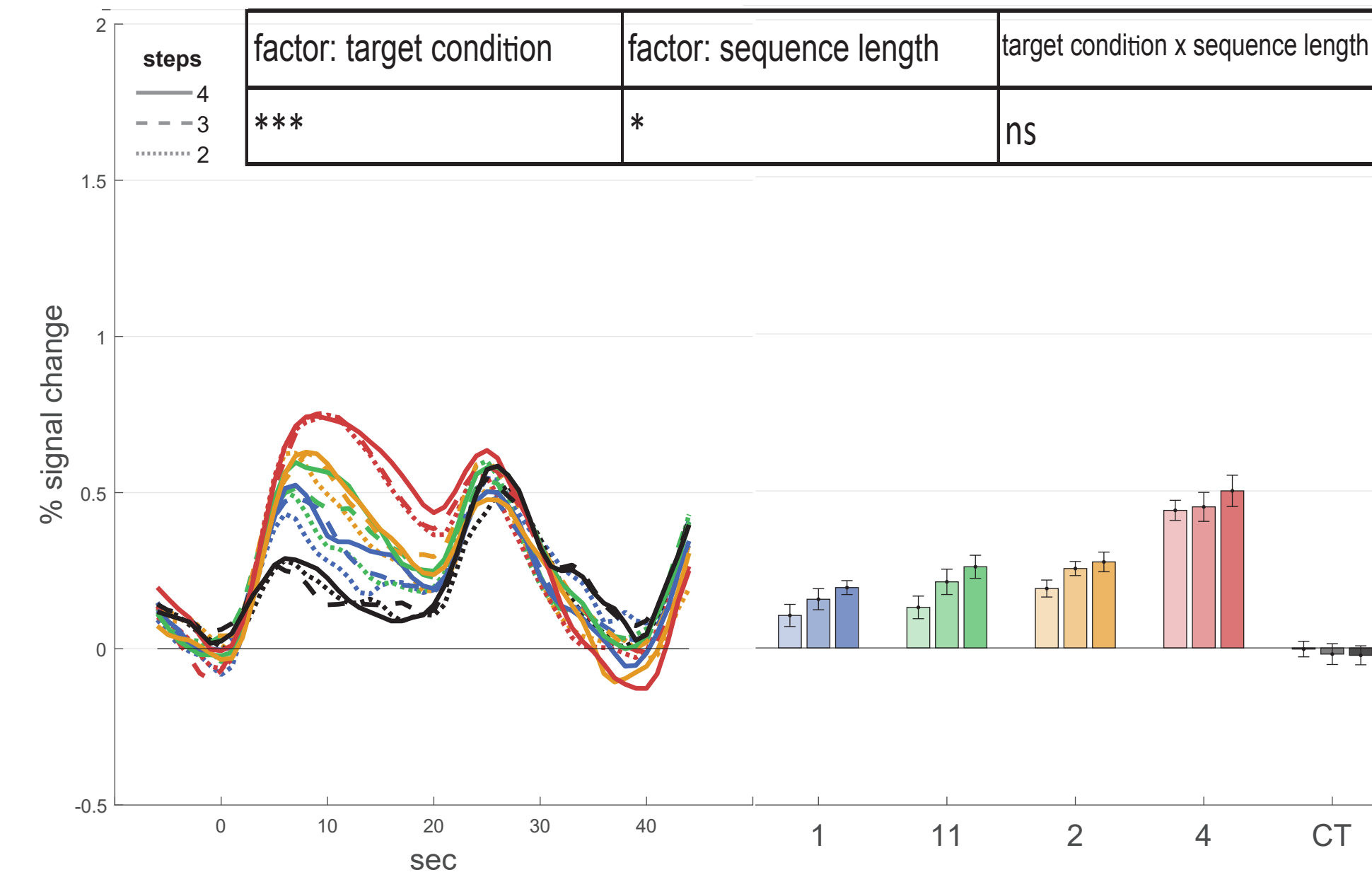

cer8r [32.4,-62.6,-52.6]

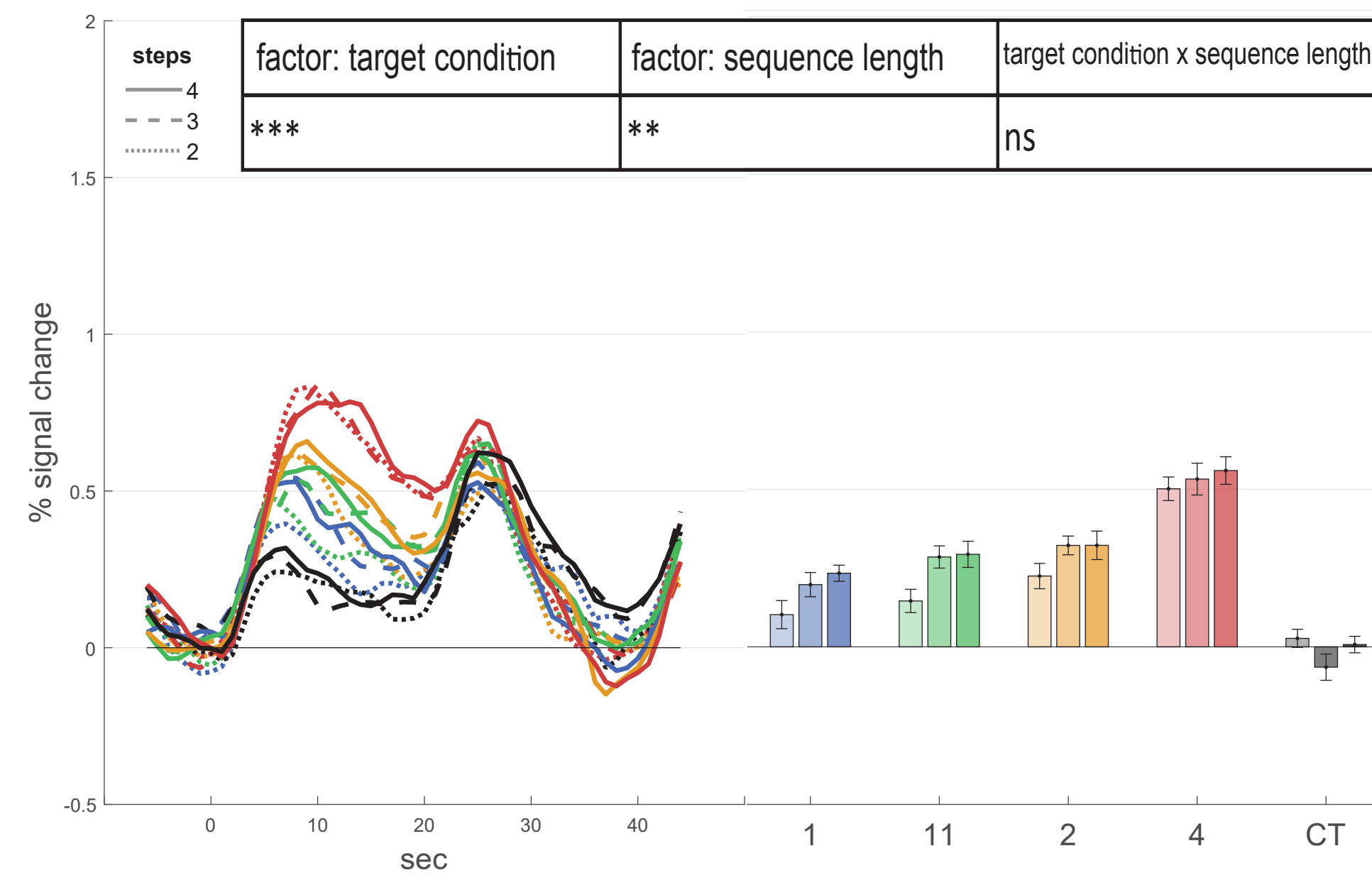

SMA [-3.6,7.9,52.1]

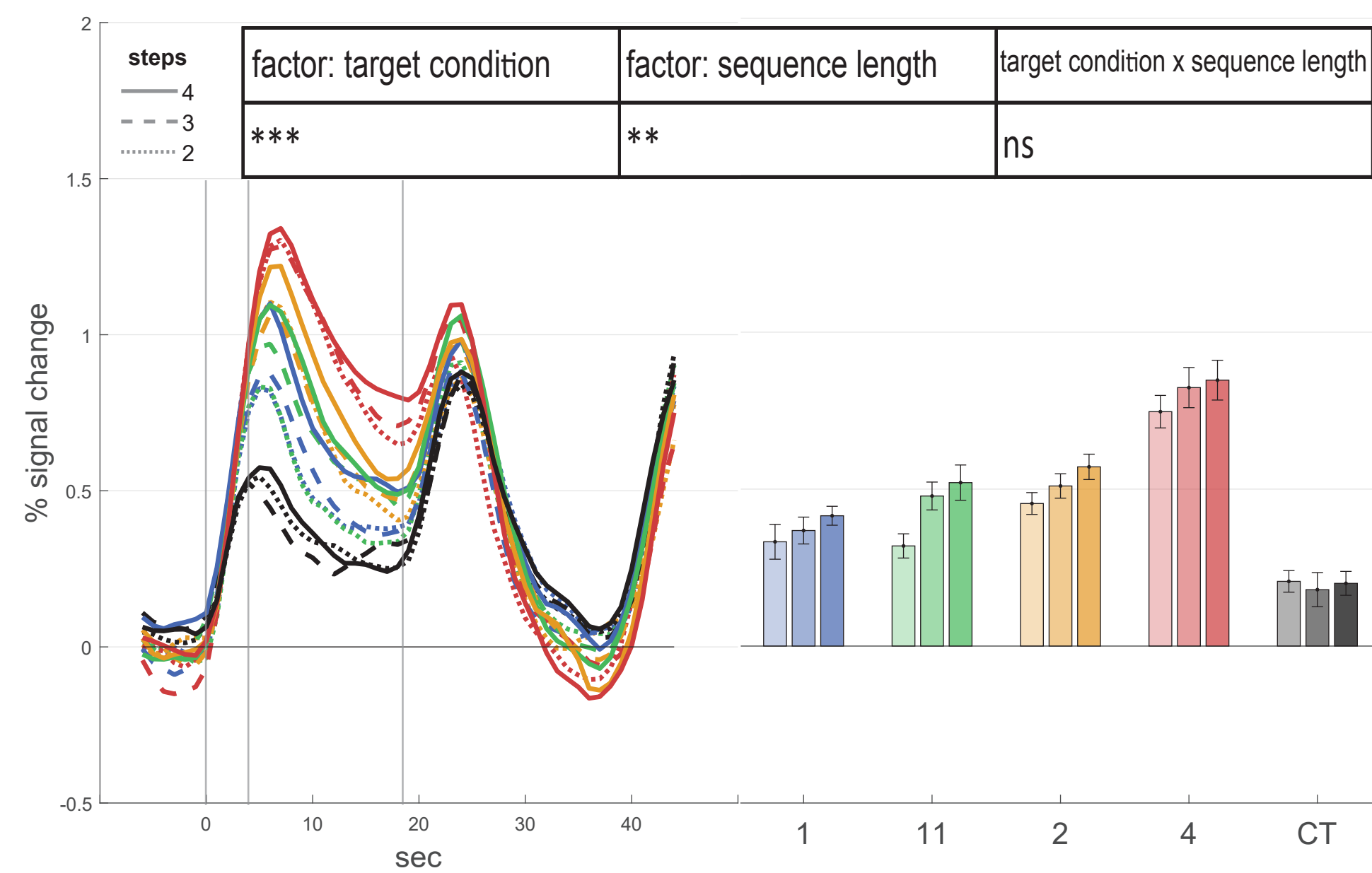

M1I [-36.8,-21.2,59.7]

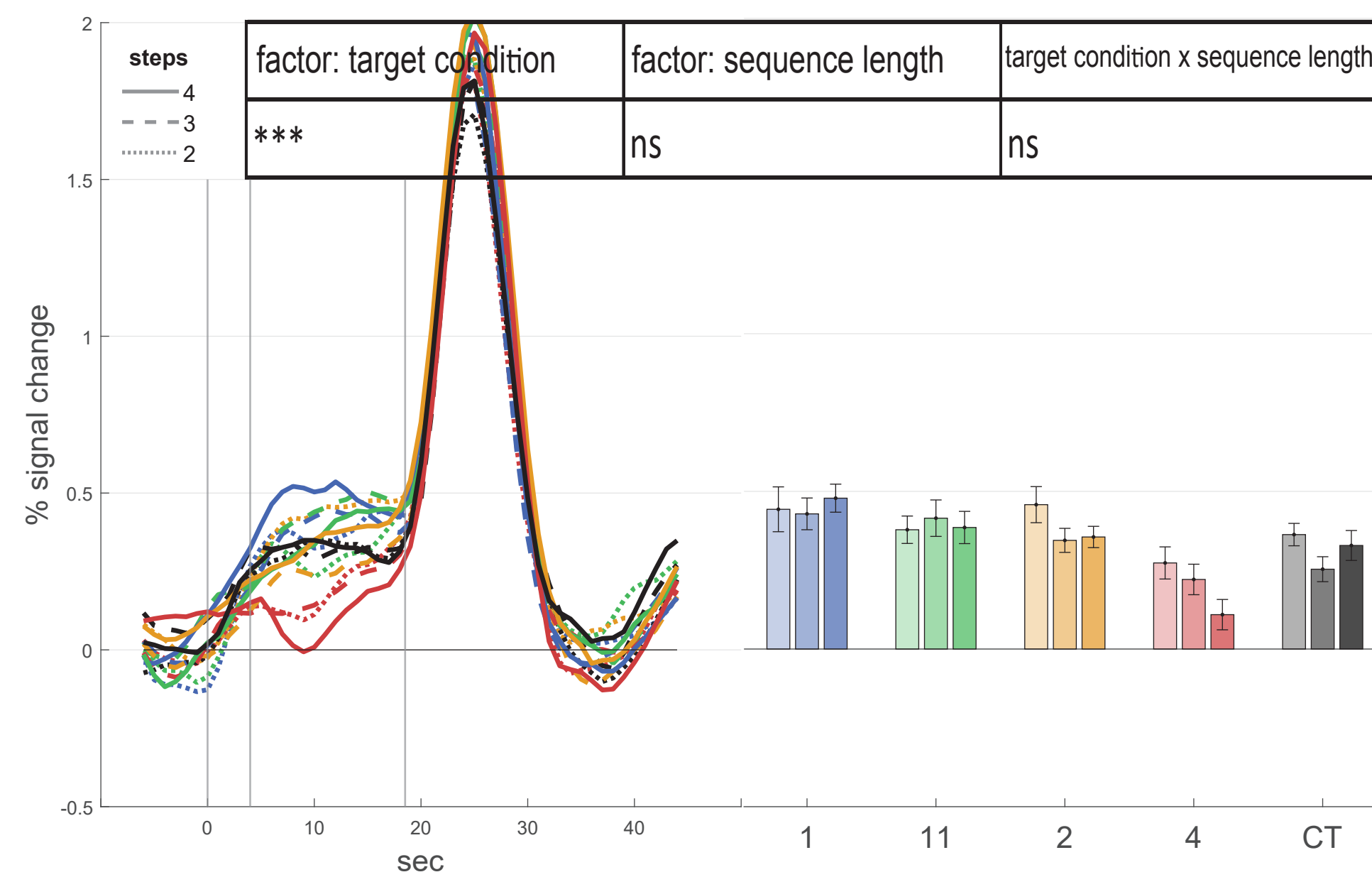

Supplement: S1 Fig — Time course of raw activity as percentage of signal change normalized to pre-stimulus baseline and delay-activity estimates (beta parameter from GLM) in each planning condition in planning-related areas (PMdl, antIPSl, DLPFCl, AICl, cer6r, cer8r) show increasing BOLD amplitude with increasing task complexity. Primary left motor cortex (M1l) as a control area shows no significant difference between conditions. We report across-subjects averages and within-subjects variance as the normalized standard error (according to [31]) and average MNI-coordinates (x, y, z in mm) for each ROI. Statistical results are indicated with *** for p < = 0.001, ** for 0.001 < p < = 0.01, * for 0.01 < p < 0.05, and ns for non-significant results (p > 0.05). (PDF) [file pcbi.1010585.s001.pdf]

**V1I [-7.1,-87,-1.3]**

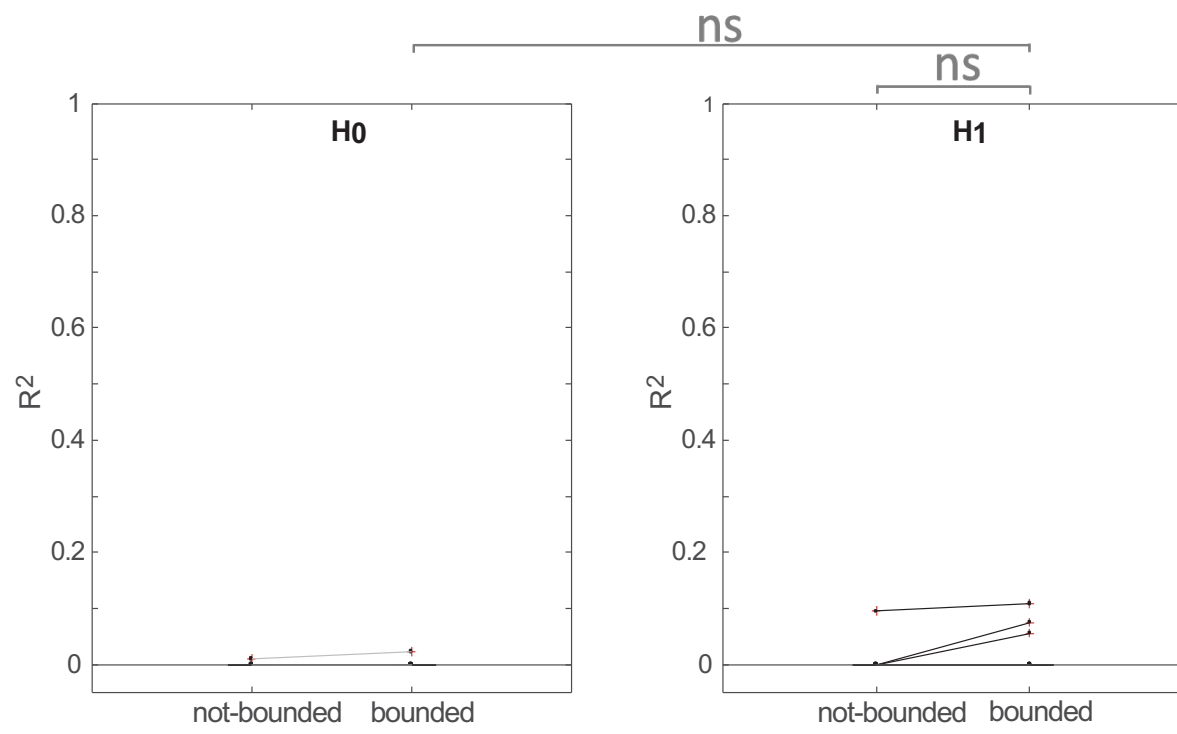

**PMdl [-21.8,-6,57.5]**

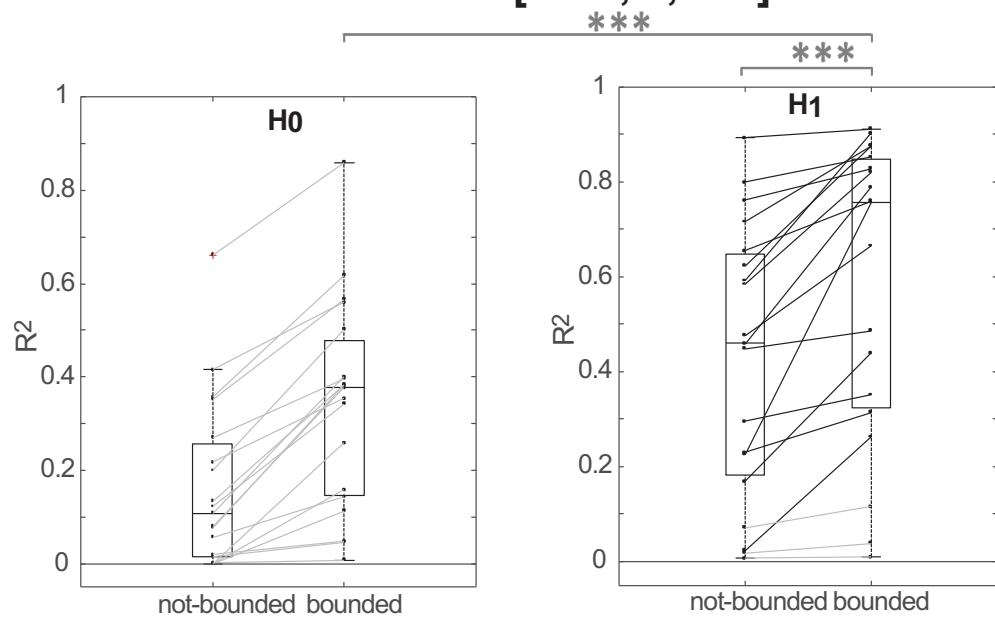

**antiPSI [-36.3,-43.6,44.7]**

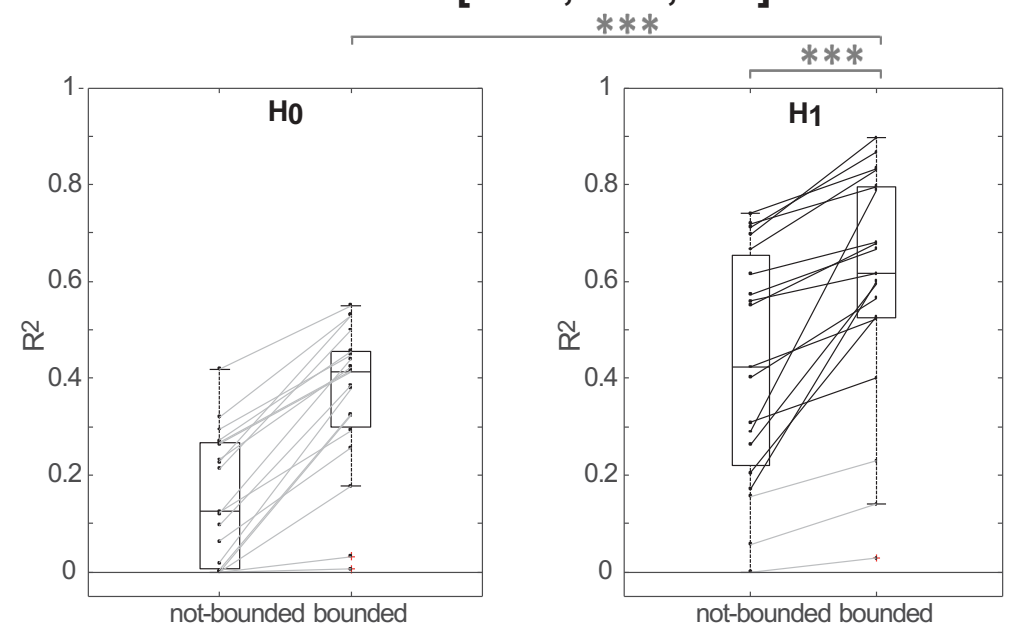

**DLPFCI [-38.4,30.2,27.8]**

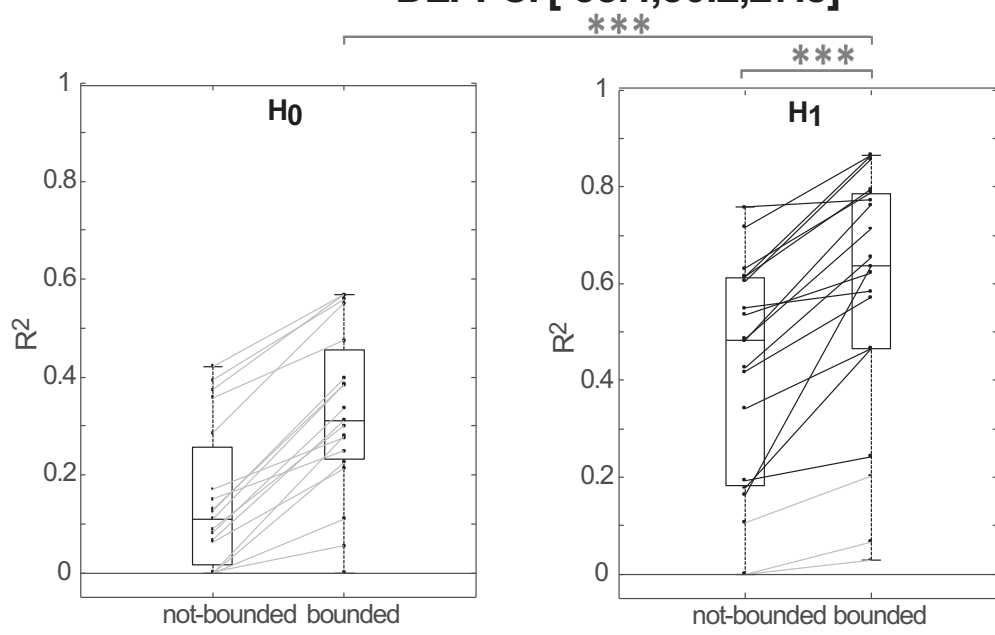

**AICI [-32.7,19.6,4.6]**

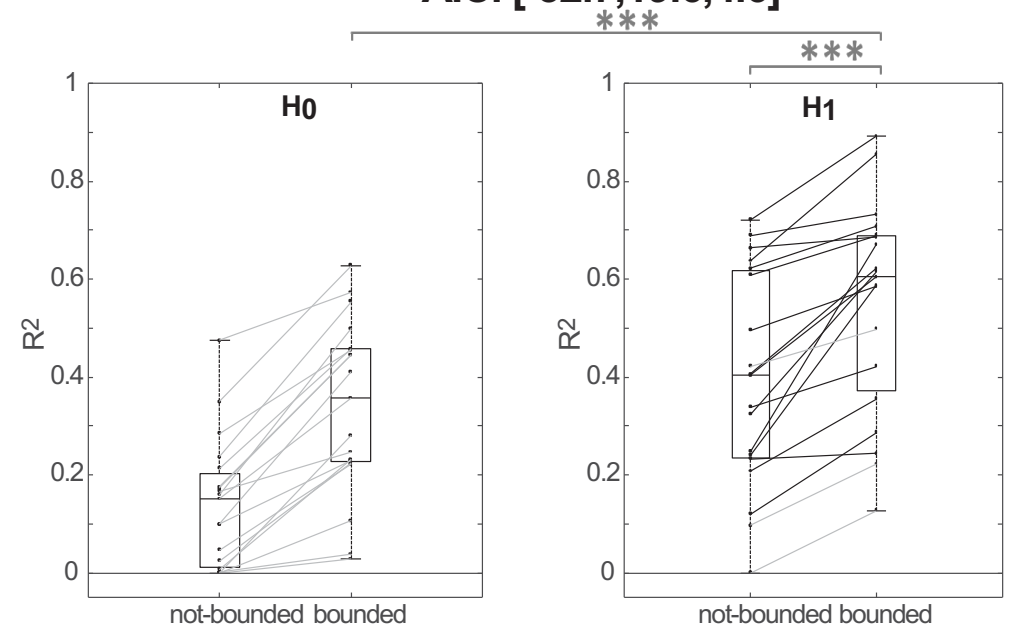

**cer6r [31.9,-57.8,-29.7]**

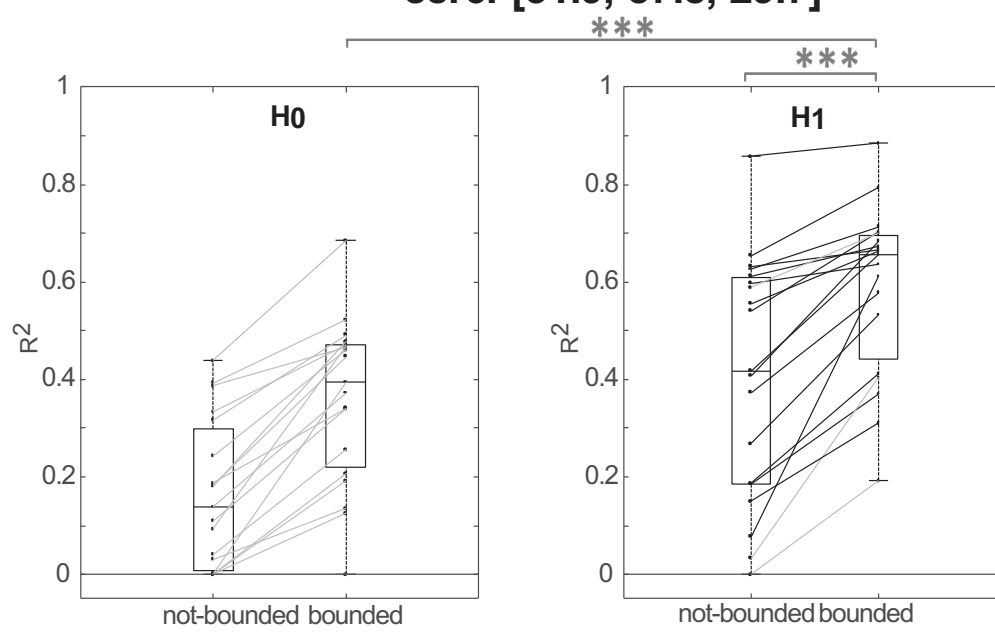

**cer8r [32.4,-62.6,-52.6]**

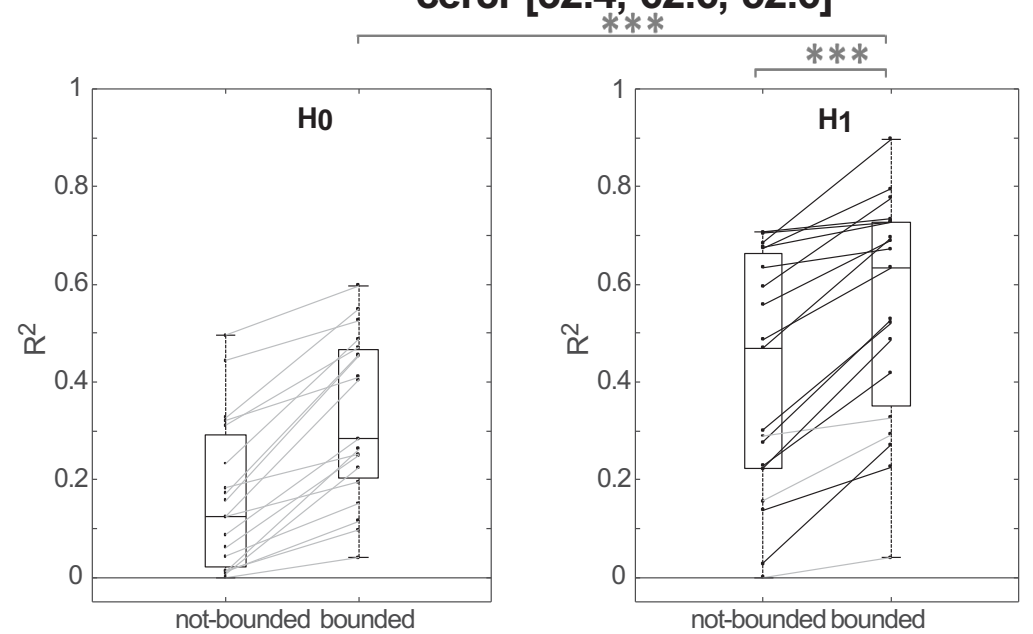

**SMA [-3.6,7.9,52.1]**

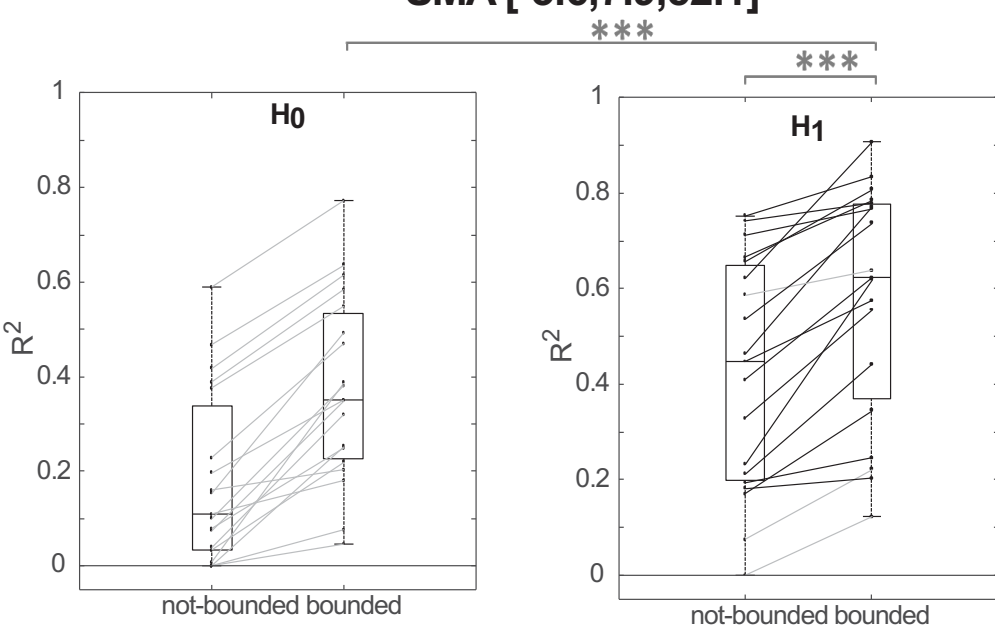

**M1I [-36.8,-21.2,59.7]**

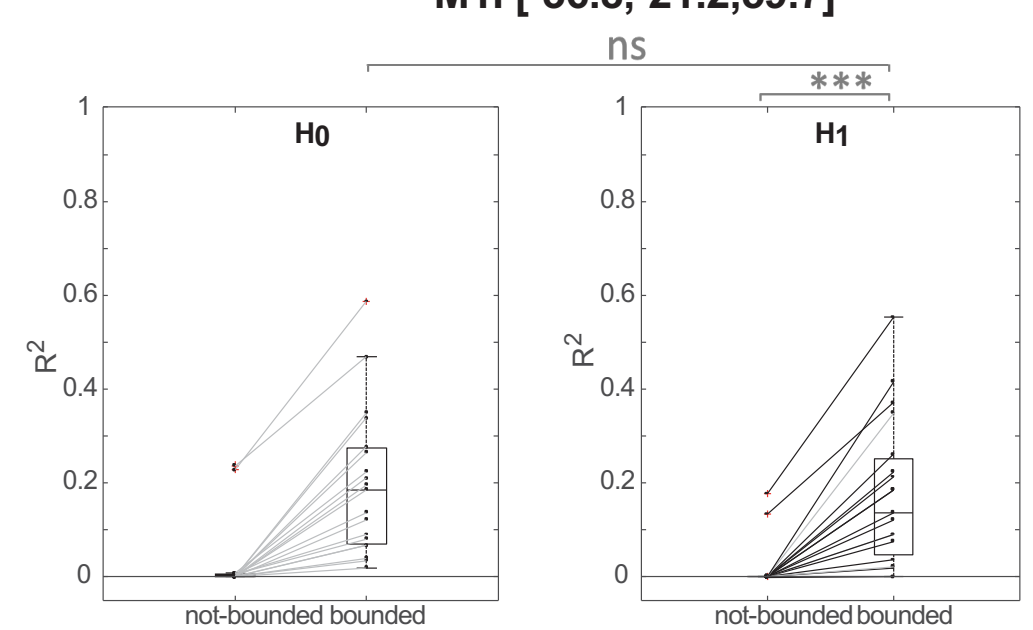

Supplement: S2 Fig — Similar to SPll, in all other planning related ROIs (PMdl, antIPSl, DLPFCl, AICl, cer6r, cer8r), a within-subject comparison of the R2-values for the two hypotheses H0 and H1 showed that the information profiles under the prospective planning hypothesis H1 provide a significantly better explanation of the fMRI modulation than the delayed planning hypothesis H0 (p < 0.0001 rmANOVA). The bounded rationality model predictions explain less of the fMRI activity modulation in control areas V1l and M1l with no significant difference between the model hypothesis (p = 0.88 and p = 0.84). We found that the correlation of measured brain activities in all other planning related ROIs (SPLl, PMdl, antIPSl, DLPFCl, AICl, cer6r, cer8r) and primary motor area (M1l) was significantly increased for model predictions with subject-individual bounds (p < 0.0001 rmANOVA) compared to an unbounded maximum capacity model. Only for control area V1l there was no significant difference between the bounded and unbounded model (p = 0.131). Statistical results of rmANOVAs are indicated with *** for p < = 0.001 and ns for non-significant results (p > 0.05). (PDF) [file pcbi.1010585.s002.pdf]

quadratic

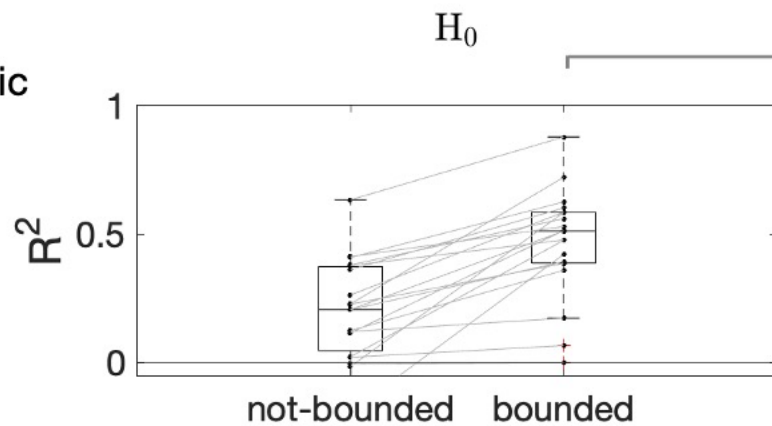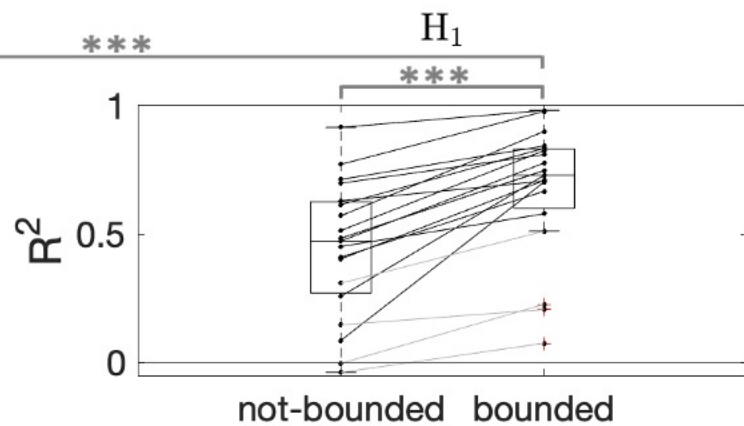

sigmoidal

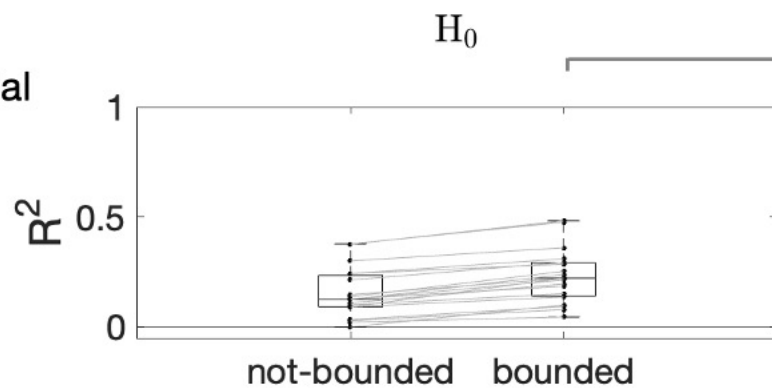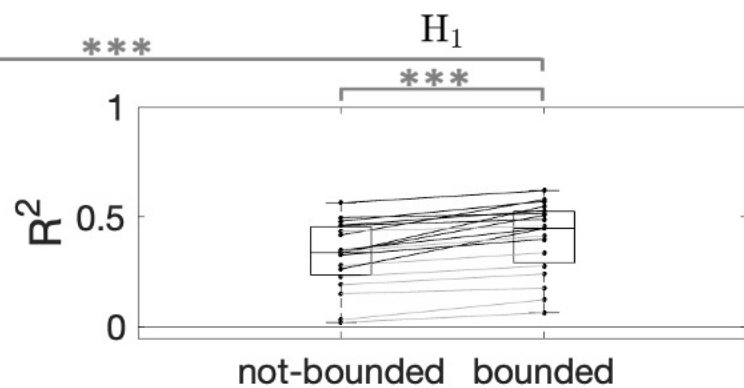

logarithmic

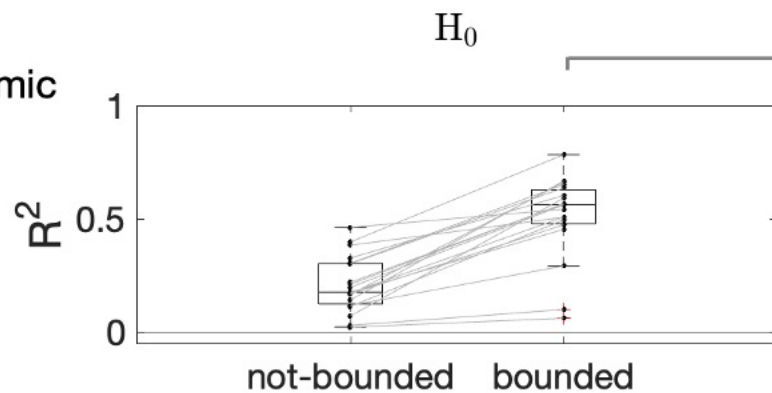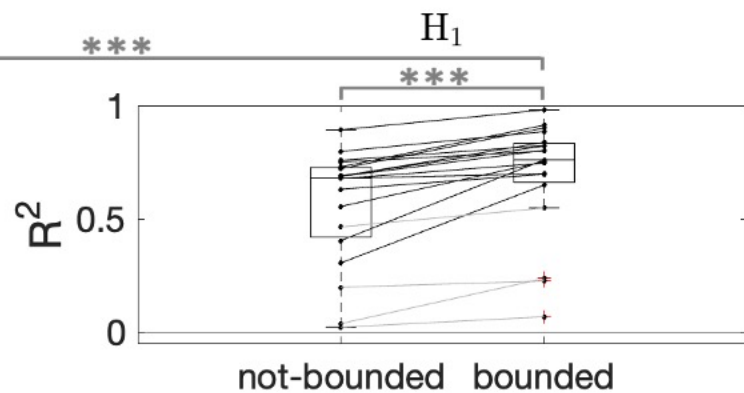

Supplement: S4 Fig — Comparison of different non-linear assumptions (quadratic, sigmoidal, logarithmic) between fMRI correlates and predicted information values for model fits of example area SPLl. Similarly to the linear assumption (compare Fig 5), R2-values of the regression analysis of the bounded rationality model under the prospective planning hypothesis H1 are significantly higher than for the delayed planning hypothesis H0 for the regressions of measured brain activities in SPLl as well as in all other planning related ROIs (also compare Supplementary S1 Table). Likewise, compared to the not-bounded H1 model hypothesis with maximum capacities (H1 not-bounded), the bounded H1 model hypothesis (H1 bounded) with subject individual capacity fits could significantly better predict measured fMRI data in SPLl as well as in all other planning related ROIs (also compare Supplementary S1 Table). Statistical results of rmANOVAs are indicated with *** for p < = 0.001. (PDF) [file pcbi.1010585.s004.pdf]
