# Supplementary material for: Bounded rational decision-making models suggest capacity-limited concurrent motor planning in human posterior parietal and frontal cortex
Source: PLoS Comput Biol. 2022 Oct 13;18(10):e1010585. doi: 10.1371/journal.pcbi.1010585 (PMC9560147; doi:10.1371/journal.pcbi.1010585)
Supplement: S3 Fig — Theoretical expected information costs I1 (left) and I2 (right) of the delayed planning hypothesis is based on model parameters determining memory and planning capacities fitted for all individual subjects (hypothesis H0 “bounded”). Histograms represent the frequency distributions of the information costs, dependent on task conditions. Information values varied between bounded model predictions regressed to subjects fMRI data compared to the model predictions in the not-bounded case (in blue). Compared to the parallel planning hypothesis H1, information I2 for action processing is lower and planning postponed until the s2-stimulus reveals the actual target location in the response phase of the experiment. (PDF) [file pcbi.1010585.s003.pdf]

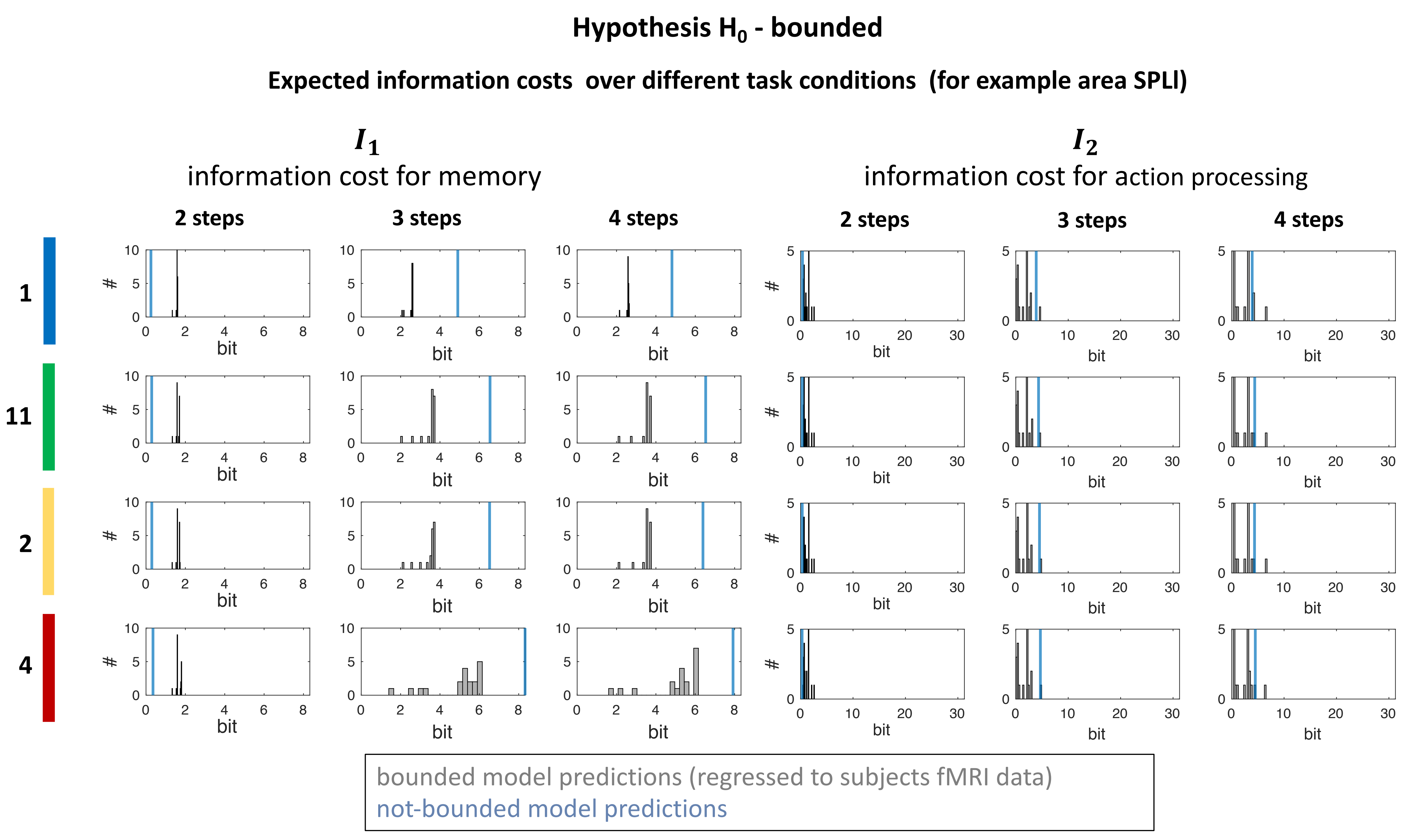

Hypothesis H0 - bounded
Expected information costs over different task conditions (for example area SPLl)
I1
information cost for memory
I2
information cost for action processing
2 steps
3 steps
4 steps
1
11
2
4
#
bit
bounded model predictions (regressed to subjects fMRI data)
not-bounded model predictions
