# Supplementary material for: Bounded rational decision-making models suggest capacity-limited concurrent motor planning in human posterior parietal and frontal cortex
Source: PLoS Comput Biol. 2022 Oct 13;18(10):e1010585. doi: 10.1371/journal.pcbi.1010585 (PMC9560147; doi:10.1371/journal.pcbi.1010585)
Supplement: S1 Table — P-values of hypothesis tests H0 vs H1 and H1 not-bounded (n-b) vs H1 bounded (b) for linear and different non-linear relation assumptions (quadratic, sigmoidal, logarithmic) between fMRI correlates and predicted information values. (PDF) [file pcbi.1010585.s005.pdf]

	linear		quadratic		sigmoidal		logarithmic	
	H_0 vs H_1	$H_1 n-b$ vs $H_1 b$	H_0 vs H_1	$H_1 n-b$ vs $H_1 b$	H_0 vs H_1	$H_1 n-b$ vs $H_1 b$	H_0 vs H_1	$H_1 n-b$ vs $H_1 b$
SPLl	0.000	0.000	0.000	0.000	0.000	0.000	0.000	0.000
PMdl	0.000	0.000	0.000	0.000	0.000	0.000	0.001	0.000
DLPFCl	0.000	0.000	0.000	0.000	0.000	0.000	0.005	0.000
antIPSl	0.000	0.000	0.000	0.000	0.000	0.000	0.002	0.000
AICl	0.000	0.000	0.000	0.029	0.000	0.000	0.133	0.000
cer6r	0.000	0.000	0.000	0.001	0.000	0.000	0.000	0.000
cer8r	0.000	0.000	0.000	0.069	0.000	0.000	0.016	0.000
SMA	0.000	0.000	0.000	0.000	0.000	0.000	0.020	0.000
V1	0.088	0.131	0.047	0.109	0.597	0.000	0.023	0.000
M1	0.084	0.000	0.077	0.007	0.004	0.000	0.671	0.000
