# Supplementary material for: Bounded rational decision-making models suggest capacity-limited concurrent motor planning in human posterior parietal and frontal cortex
Source: PLoS Comput Biol. 2022 Oct 13;18(10):e1010585. doi: 10.1371/journal.pcbi.1010585 (PMC9560147; doi:10.1371/journal.pcbi.1010585)
Supplement: S2 Table — R2-values of the linear regression analysis of the bounded rationality model under the hypothesis H0 and H1. Group median with lower and upper quartiles of the bounded model assumptions (best fitting information capacities of individual subjects) are compared to the not-bounded case. (PDF) [file pcbi.1010585.s006.pdf]

	H_0 - not-bounded			H_1 - not-bounded		
	median	75% quantile	25% quantile	median	75% quantile	25% quantile
SPLl	0.12	0.28	0.04	0.51	0.64	0.34
PMdl	0.12	0.27	0.01	0.46	0.65	0.18
DLPFCl	0.10	0.25	0.01	0.48	0.61	0.18
antIPSl	0.11	0.26	0.01	0.42	0.65	0.22
AICl	0.15	0.21	0.01	0.40	0.62	0.23
cer6r	0.13	0.31	0.01	0.42	0.61	0.19
cer8r	0.12	0.28	0.02	0.47	0.66	0.22
SMA	0.09	0.32	0.04	0.45	0.65	0.20
V1	0.00	0.00	0.00	0.00	0.00	0.00
M1	0.00	0.00	0.00	0.00	0.00	0.00

	H_0 - bounded			H_1 - bounded		
	median	75% quantile	25% quantile	median	75% quantile	25% quantile
SPLl	0.41	0.52	0.28	0.74	0.83	0.58
PMdl	0.38	0.48	0.15	0.76	0.85	0.32
DLPFCl	0.31	0.46	0.23	0.64	0.78	0.47
antIPSl	0.41	0.45	0.30	0.62	0.79	0.52
AICl	0.36	0.46	0.23	0.60	0.69	0.37
cer6r	0.39	0.47	0.22	0.66	0.70	0.44
cer8r	0.28	0.47	0.20	0.63	0.73	0.35
SMA	0.35	0.53	0.23	0.62	0.78	0.37
V1	0.00	0.00	0.00	0.00	0.00	0.00
M1	0.19	0.27	0.07	0.14	0.25	0.05
