# Supplementary material for: Bounded rational decision-making models suggest capacity-limited concurrent motor planning in human posterior parietal and frontal cortex
Source: PLoS Comput Biol. 2022 Oct 13;18(10):e1010585. doi: 10.1371/journal.pcbi.1010585 (PMC9560147; doi:10.1371/journal.pcbi.1010585)
Supplement: S3 Table — Expected information E[I1] over all experimental conditions for all 19 subjects, measured in bits. For maximal capacity, information E[I1]=4.428 bits. (PDF) [file pcbi.1010585.s007.pdf]

subjects	$SPLl$	$PMdl$	$DLPFCl$	$antIPS$	$AICl$	$cer6r$	$cer8r$	SMA	$V1l$	$M1l$
1	3.24	3.24	3.06	3.24	3.08	3.25	3.24	3.08	1.49	1.67
2	2.97	2.97	2.64	3	2.97	2.64	1.82	2.97	1.77	2.97
3	3.03	3.03	3.03	3.03	3.08	3.06	3.25	3.03	1.49	3.09
4	3.25	3.25	3.24	3.25	3.08	3.25	3.24	3.25	4.62	1.67
5	3.08	3.08	3.25	3.25	3.25	3.25	3.25	2.64	1.77	2.97
6	3.03	3.03	1.38	1.38	1.38	1.38	2.97	1.38	1.43	1.49
7	3.24	3.09	3.24	3.11	3.09	3.08	3.23	3.03	1.53	1.51
8	3.03	3.06	3.06	3.08	3.08	3.08	3.24	3.09	1.77	3.09
9	1.77	2.78	1.77	1.72	3.09	1.73	1.72	3.09	1.77	1.74
10	2.65	3.25	3.16	3.16	3.17	2.65	3.22	3.15	1.96	2.97
11	3.25	3.03	3.18	3.25	3.24	3.21	3.25	3.18	1.77	1.96
12	3.09	3.06	3.06	3.1	3.25	2.43	3.08	3.08	1.77	1.82
13	3.03	3.09	3.03	3.03	3.06	3.03	3.25	3.03	1.77	1.8
14	2.3	2.78	3.19	3.12	3.09	1.54	3.12	3.09	1.54	3.09
15	3.24	3.19	3.19	3.24	1.67	1.8	3.17	1.67	1.77	1.67
16	3.24	3.24	3.24	3.24	3.24	3.25	3.24	3.18	1.54	1.72
17	3.24	3.24	3.24	3.24	3.24	3.24	3.24	3.24	1.77	1.7
18	2.97	3.09	2.97	2.97	3.07	3.24	3.08	2.97	1.77	1.72
19	2.62	2.82	2.62	2.62	2.88	2.5	2.24	2.82	1.77	2.97
mean	2.96	3.07	2.92	2.95	2.95	2.72	2.99	2.89	1.85	2.19
