# Supplementary material for: Bounded rational decision-making models suggest capacity-limited concurrent motor planning in human posterior parietal and frontal cortex
Source: PLoS Comput Biol. 2022 Oct 13;18(10):e1010585. doi: 10.1371/journal.pcbi.1010585 (PMC9560147; doi:10.1371/journal.pcbi.1010585)
Supplement: S4 Table — Expected information E[I2] over all experimental conditions for all 19 subjects, measured in bits. For maximal capacity, information E[I2]=2.994 bits. (PDF) [file pcbi.1010585.s008.pdf]

subjects	$SPLl$	$PMdl$	$DLPFCl$	$antIPS$	$AICl$	$cer6r$	$cer8r$	SMA	$V1l$	$M1l$
1	2.32	2.32	0.7	2.32	1.01	2.42	2.32	0.93	2.65	2.97
2	0.4	0.4	0.36	0.53	0.4	0.36	0.61	0.4	2.57	0.4
3	0.53	0.53	0.53	0.53	0.93	0.64	2.42	0.53	2.65	2.39
4	2.42	2.42	2.32	2.42	1.01	2.42	2.32	2.42	2.88	2.97
5	0.93	0.93	2.42	2.42	2.42	2.42	2.42	0.36	2.57	0.4
6	0.53	0.53	0.42	0.42	0.42	0.42	0.4	0.42	2.52	3.09
7	2.32	2.39	2.32	2.61	2.39	0.93	2.3	0.53	2.62	3.03
8	0.53	0.7	0.7	0.93	0.93	0.93	2.32	2.39	2.57	2.39
9	2.57	2.5	2.57	3.16	2.39	3.04	3.16	2.39	2.57	2.98
10	0.46	2.4	2.38	2.38	2.4	0.46	2.37	2.39	2.76	0.4
11	2.42	0.53	2.37	2.42	2.32	2.54	2.42	2.37	2.57	2.85
12	2.39	0.7	0.7	2.42	2.42	0.22	0.93	0.93	2.57	0.61
13	0.53	2.39	0.53	0.53	0.64	0.53	2.42	0.53	2.57	3.1
14	3.38	2.5	2.36	2.64	2.39	2.85	2.64	2.39	2.85	2.39
15	2.32	2.42	2.36	2.32	2.97	3.1	2.44	2.97	2.57	2.97
16	2.32	2.32	2.32	2.32	2.32	2.42	2.42	2.37	2.85	3.16
17	2.32	2.32	2.32	2.32	2.32	2.42	2.42	2.42	2.57	2.59
18	0.4	2.39	0.4	0.4	1.37	2.42	2.63	0.4	2.57	3.16
19	3.19	3.04	3.19	3.19	3.1	1.41	3.42	3.04	2.57	0.4
mean	1.7	1.77	1.65	1.91	1.8	1.68	2.23	1.59	2.63	2.22
