# Supplementary material for: Bounded rational decision-making models suggest capacity-limited concurrent motor planning in human posterior parietal and frontal cortex
Source: PLoS Comput Biol. 2022 Oct 13;18(10):e1010585. doi: 10.1371/journal.pcbi.1010585 (PMC9560147; doi:10.1371/journal.pcbi.1010585)
Supplement: S5 Table — Expected information E[I1] over all experimental conditions for all 19 subjects, measured in bits. For maximal capacity, information E[I1]=4.428 bits. (PDF) [file pcbi.1010585.s009.pdf]

subjects	$SPLl$	$PMdl$	$DLPFCl$	$antIPS$	$AICl$	$cer6r$	$cer8r$	SMA	$V1l$	$M1l$
1	2.86	1.74	2.6	1.69	1.67	3.14	2.44	2.36	1.49	1.8
2	2.78	2.78	2.73	2.97	2.97	1.82	1.82	2.78	1.77	1.82
3	3.08	3.07	3.08	3.09	3.1	3.06	3.09	2.82	2.12	1.49
4	3.2	3.14	3.1	3.17	3.18	3.12	3.1	3.17	4.94	1.67
5	3.17	3.16	2.57	2.27	2.12	2.25	2.41	2.32	1.77	1.82
6	3.05	3.07	3.07	3.06	3.08	3.09	2.77	3.08	1.43	1.49
7	3.09	2.97	3.08	1.69	3.18	1.68	1.69	2.65	1.53	1.51
8	3.1	3.09	3.1	3.12	3.1	3.16	3.1	3.08	1.77	1.82
9	2.97	1.72	2.97	2.97	2.12	1.73	1.72	3.01	1.77	1.74
10	1.8	1.8	1.8	1.97	3.19	2.24	1.82	3.15	1.96	2.12
11	3.11	2.83	3.04	1.89	4.8	2.42	3.16	3.11	1.77	1.96
12	2.7	2.53	3.09	1.67	2.8	2.83	3.03	3.09	1.77	1.82
13	3.01	3.03	3.11	3.05	3.09	3.1	3.17	3.1	1.77	1.8
14	2.3	1.7	3.09	1.7	3.1	2.97	1.7	2.97	1.54	1.67
15	1.69	1.69	1.7	1.69	1.8	1.8	1.67	1.8	1.77	1.67
16	3.08	3.08	3.08	3.01	3.1	2.43	1.77	1.7	2.25	1.72
17	1.68	1.68	1.74	2.44	1.7	1.77	4.8	1.7	1.77	1.72
18	2.73	2.65	2.12	2.7	1.8	1.7	1.69	2.78	1.77	1.72
19	1.91	2.62	2.62	2.47	2.73	2.62	2.08	2.62	1.77	2.05
mean	2.7	2.54	2.72	2.45	2.77	2.47	2.48	2.7	1.93	1.76
