# Supplementary material for: Bounded rational decision-making models suggest capacity-limited concurrent motor planning in human posterior parietal and frontal cortex
Source: PLoS Comput Biol. 2022 Oct 13;18(10):e1010585. doi: 10.1371/journal.pcbi.1010585 (PMC9560147; doi:10.1371/journal.pcbi.1010585)
Supplement: S6 Table — Expected information E[I2] over all experimental conditions for all 19 subjects, measured in bits. For maximal capacity, information E[I2]=14.109 bits. (PDF) [file pcbi.1010585.s010.pdf]

subjects	$SPLl$	$PMdl$	$DLPFCl$	$antIPS$	$AICl$	$cer6r$	$cer8r$	SMA	$V1l$	$M1l$
1	12	12	11.92	12.16	12.71	12.66	12.17	12.01	12.75	13.65
2	11.36	11.36	12.19	10.02	3.01	4.77	4.77	11.36	12.46	4.77
3	9.96	7.65	6.59	6.93	12.19	8.02	12.12	11.82	1.85	12.75
4	12.25	11.9	11.9	11.99	12.17	11.84	11.9	11.93	14.95	12.71
5	12.27	12.25	12.22	12.01	12.62	14.43	12.12	12.42	12.46	4.77
6	6.03	6.35	4.96	6.43	7.27	12.12	8.87	6.59	12.45	13.62
7	11.76	3.01	11.86	12.3	11.86	12.09	12.16	11.79	12.64	13.47
8	12.11	11.76	11.96	11.83	11.9	12.1	11.85	11.86	12.46	4.77
9	3.01	13.79	3.01	3.01	1.85	13.49	13.79	7.46	12.46	13.38
10	13.4	13.4	13.4	14.34	12.26	14.24	12.31	12.15	12.89	1.85
11	12.68	12.05	13.66	12.9	14.64	12.07	12.28	13.8	12.46	13.1
12	11.81	11.79	9.87	12.71	11.83	11.95	10.92	9.25	12.46	4.77
13	5.04	3.65	12	9.58	8.69	12.11	12.04	12.11	12.46	13.65
14	14.12	12.57	11.76	12.57	11.96	3.01	12.57	3.01	13.11	12.71
15	12.16	12.16	12.57	12.13	13.65	13.65	12.71	13.65	12.46	12.71
16	11.86	11.86	11.86	11.97	11.85	12.08	12.9	12.57	5.04	13.79
17	12.17	12.17	13.38	12.17	12.57	12.9	14.64	12.57	12.46	13.79
18	12.19	3.48	1.85	12.11	13.65	12.57	12.3	11.36	12.46	13.79
19	11.91	12.98	13.9	13.9	12.19	12.98	5.11	12.98	12.46	2.31
mean	10.95	10.32	10.57	11.11	10.99	11.53	11.45	11.09	11.73	10.33
