# Supplementary material for: Bounded rational decision-making models suggest capacity-limited concurrent motor planning in human posterior parietal and frontal cortex
Source: PLoS Comput Biol. 2022 Oct 13;18(10):e1010585. doi: 10.1371/journal.pcbi.1010585 (PMC9560147; doi:10.1371/journal.pcbi.1010585)
Supplement: S7 Table — Expected utility E[U] over all experimental conditions for all 19 subjects. (PDF) [file pcbi.1010585.s011.pdf]

subjects	$SPLl$	$PMdl$	$DLPFCl$	$antIPS$	$AICl$	$cer6r$	$cer8r$	SMA	$V1l$	$M1l$
1	1	1	0.56	1	0.65	1	1	0.63	0.81	0.75
2	0.43	0.43	0.43	0.48	0.43	0.49	0.37	0.43	0.86	0.43
3	0.48	0.48	0.48	0.48	0.63	1	0.48	0.48	0.81	1
4	1	1	1	1	0.65	1	1	1	1	0.75
5	0.63	0.63	1	1	1	1	1	0.43	0.86	0.43
6	0.48	0.48	0.48	0.48	0.48	0.48	0.48	0.48	0.79	0.8
7	1	1	1	0.99	1	0.67	0.43	0.48	0.82	0.8
8	0.48	0.56	0.56	0.63	0.63	1	1	1	0.86	1
9	0.86	0.87	0.86	0.83	1	0.85	0.48	1	0.86	0.84
10	0.47	1	1	1	1	0.37	0.56	1	0.89	0.43
11	1	0.48	1	1	1	1	1	1	0.86	0.88
12	1	0.56	0.56	1	1	0.56	0.48	0.63	0.86	0.54
13	0.48	1	0.48	0.48	0.53	1	0.53	0.48	0.86	0.86
14	0.91	0.87	1	1	1	1	1	1	0.82	1
15	1	1	1	1	0.75	0.85	0.75	0.75	0.86	0.75
16	1	1	1	1	1	1	1	1	0.82	0.83
17	1	1	1	1	1	1	1	1	0.86	0.85
18	0.43	1	0.43	0.43	0.76	1	0.43	0.43	0.86	0.83
19	0.95	0.97	0.95	0.95	0.98	0.99	0.59	0.97	0.86	0.43
mean	0.77	0.81	0.78	0.83	0.81	0.86	0.71	0.75	0.86	0.75
