# Supplementary material for: Bounded rational decision-making models suggest capacity-limited concurrent motor planning in human posterior parietal and frontal cortex
Source: PLoS Comput Biol. 2022 Oct 13;18(10):e1010585. doi: 10.1371/journal.pcbi.1010585 (PMC9560147; doi:10.1371/journal.pcbi.1010585)
Supplement: S8 Table — Expected utility E[U] over all experimental conditions for all 19 subjects. (PDF) [file pcbi.1010585.s012.pdf]

subjects	$SPLl$	$PMdl$	$DLPFCl$	$antIPS$	$AICl$	$cer6r$	$cer8r$	SMA	$V1l$	$M1l$
1	0.98	0.86	0.96	0.85	0.75	1	1	0.95	0.81	0.86
2	0.87	0.87	0.87	0.85	0.43	0.49	0.79	0.87	0.86	0.54
3	0.89	0.76	0.7	0.72	1	0.87	0.65	0.96	0.33	0.81
4	1	1	1	1	1	1	1	1	0.99	0.75
5	1	1	0.96	0.94	0.91	0.94	0.96	0.94	0.86	0.54
6	0.65	0.68	0.58	0.67	0.74	0.56	0.48	0.7	0.79	0.8
7	1	0.43	1	0.85	1	0.86	0.75	0.97	0.82	0.8
8	1	1	1	1	1	1	1	1	0.86	0.54
9	0.43	0.83	0.43	0.43	0.33	0.84	0.81	0.73	0.86	0.84
10	0.86	0.86	0.86	0.88	1	0.9	0.9	1	0.89	0.33
11	0.99	0.99	0.99	0.88	0.99	1	0.97	0.99	0.86	0.88
12	0.97	0.96	0.88	0.75	0.97	0.53	0.53	0.85	0.86	0.54
13	0.58	0.48	1	0.86	0.82	0.96	0.84	1	0.86	0.86
14	0.91	0.85	1	0.85	1	0.85	1	0.43	0.82	0.75
15	0.85	0.85	0.85	0.85	0.86	0.86	0.54	0.86	0.86	0.75
16	1	1	1	0.99	1	0.95	1	0.85	0.58	0.83
17	0.85	0.85	0.84	0.95	0.85	0.92	0.92	0.85	0.86	0.83
18	0.87	0.47	0.33	0.91	0.86	0.99	0.97	0.87	0.86	0.83
19	0.89	0.92	0.95	0.93	0.87	0.99	0.59	0.92	0.86	0.37
mean	0.87	0.82	0.85	0.85	0.86	0.87	0.83	0.88	0.82	0.71
