# Supplementary material for: Bounded rational decision-making models suggest capacity-limited concurrent motor planning in human posterior parietal and frontal cortex
Source: PLoS Comput Biol. 2022 Oct 13;18(10):e1010585. doi: 10.1371/journal.pcbi.1010585 (PMC9560147; doi:10.1371/journal.pcbi.1010585)
Supplement: S9 Table — MNI coordinates of the group coordinates and average spatial dispersion of the ROI centers on individual subjects around the group ROI coordinates. (PDF) [file pcbi.1010585.s013.pdf]

	group coordinates			± difference to individual coordinates		
	x	y	z	x	y	z
SPLl	−9	−69	57	6.63	−2.68	−0.63
SPLr	9	−63	54	−9	3.47	−3.63
PMdl	−18	−3	63	3.79	3	5.53
PMdr	27	−6	60	1.58	−2.68	4.58
DLPFCl	−36	30	24	2.37	−0.16	−3.79
DLPFCr	36	45	27	−1.26	9.79	−2.84
antIPSl	−39	−45	45	−2.68	−1.42	0.32
antIPSr	36	−42	42	−0.16	−0.79	−2.84
AICl	−33	15	6	−0.32	−4.58	1.42
AICr	36	18	9	0.95	−2.37	5.21
SMA	9	6	54	12.63	−1.89	1.89
V1l	0	−78	3	7.11	9	4.26
M1l	−42	−15	60	−5.21	6.16	0.32
cer6l	−27	−57	−33	1.89	1.26	−2.68
cer6r	33	−57	−30	1.11	0.79	−0.32
cer8l	−33	−60	−48	−0.63	1.58	4.89
cer8r	33	−60	−48	0.63	2.58	4.58
