# Supplementary material for: Bounded rational decision-making models suggest capacity-limited concurrent motor planning in human posterior parietal and frontal cortex
Source: PLoS Comput Biol. 2022 Oct 13;18(10):e1010585. doi: 10.1371/journal.pcbi.1010585 (PMC9560147; doi:10.1371/journal.pcbi.1010585)
Supplement: S10 Table — Predictive ability of the suggested models was evaluated using leave-one-out cross-validation. Multi-linear regression of the measured fMRI modulation and the predicted information values I1 and I2 was performed on data with 11 out of 12 conditions in a repeated manner and regression parameters used to predict the fMRI activity in the left-out condition. In all brain areas except the control areas M1l and V1l, the prediction error under the concurrent prospective planning hypothesis H1 is significantly lower than the prediction error under the delayed planning hypothesis H0. Similarly, under the prospective planning hypothesis H1 the “bounded” models with subject-individual best-fitting information capacities (H1 b) have a significantly lower prediction error in the cross-validation than the “not-bounded” models with maximum capacity (H1 n-b). Non-parametric pairwise comparison was performed using Wilcoxon signrank test with an Bonferroni corrected significance level (p = 0.00294), when testing for all the 17 ROIs (left and right hemispheres, see Methods). (PDF) [file pcbi.1010585.s014.pdf]

	H_0 vs H_1	$H_1 n-b$ vs $H_1 b$
SPLl	0.00290	0.00290
PMdl	0.00097	0.00097
DLPFCl	0.00223	0.00223
antIPSl	0.00062	0.00062
AICl	0.00084	0.00084
cer6r	0.00016	0.00016
cer8r	0.00062	0.00062
SMA	0.00054	0.00054
V1	0.25000	0.25000
M1	0.04862	0.04862
